# Transferrin Coated Nanoparticles: Study of the Bionano Interface in Human Plasma

**DOI:** 10.1371/journal.pone.0040685

**Published:** 2012-07-19

**Authors:** Andrzej S. Pitek, David O’Connell, Eugene Mahon, Marco P. Monopoli, Francesca Baldelli Bombelli, Kenneth A. Dawson

**Affiliations:** 1 Centre for BioNano Interactions, School of Chemistry and Chemical Biology, University College Dublin, Dublin, Ireland; 2 Conway Institute for Biomolecular and Biomedical Research, University College Dublin, Dublin, Ireland; 3 School of Pharmacy, University of East Anglia, Norwich, United Kingdom; RMIT University, Australia

## Abstract

It is now well established that the surface of nanoparticles (NPs) in a biological environment is immediately modified by the adsorption of biomolecules with the formation of a protein corona and it is also accepted that the protein corona, rather than the original nanoparticle surface, defines a new biological identity. Consequently, a methodology to effectively study the interaction between nanomaterials and the biological corona encountered within an organism is a key objective in nanoscience for understanding the impact of the nanoparticle-protein interactions on the biological response *in vitro* and *in vivo*. Here, we outline an integrated methodology to address the different aspects governing the formation and the function of the protein corona of polystyrene nanoparticles coated with Transferrin by different strategies. Protein-NP complexes are studied both *in situ* (in human plasma, full corona FC) and after washing (hard corona, HC) in terms of structural properties, composition and second-order interactions with protein microarrays. Human protein microarrays are used to effectively study NP-corona/proteins interactions addressing the growing demand to advance investigations of the extrinsic function of corona complexes. Our data highlight the importance of this methodology as an analysis to be used in advance of the application of engineered NPs in biological environments.

## Introduction

Today nanomaterials are widely used in medicine with an increasing concern about their possible long-term undesirable effects on humans. Even with major progress in effective application of such nanomaterials, their behaviour *in vivo* remains poorly characterized. To evaluate the potential impact of engineered nanomaterials on human health and to improve their performance, it is essential to study their behaviour in biological media [1]–[3].

It is now well established that the surface of nanoparticles (NPs) in a biological environment is immediately modified by the adsorption of biomolecules such as proteins, lipids, glycans, etc. with the formation of the so-called protein corona [4]–[9]. It is also accepted that the protein corona, rather than the pristine NP itself, defines the biological identity of NPs [7], [10]–[14]. The physicochemical properties of such particle-protein complexes are different than those of the formulated particles and the nature of adsorbed proteins will determine the possible interactions in the biological environment. Clearly, the understanding of the protein-nanoparticle interaction represents a crucial step for predicting NPs biodistribution *in vivo* with obvious implications for clearance by macrophage uptake and possible toxicity due to activation of defence responses, such as clotting or complement [7], [15]–[17]. Consequently, the need of a new common methodology to effectively study the interaction between nanomaterials and the biological corona encountered within an organism is a key objective in nanoscience. In the literature, there are countless examples of newly engineered nanomaterials aiming either to target specific body compartments [18]–[21], or to work as diagnostic and therapeutic agents [13], [22]–[28]. However, these nano-objects are often not fully characterized in the relevant biological environment, where they are normally distinguished by a new biological interface interacting with the living matter. The lack of definition of the real biological surface of the nanomaterials *in situ* can be an obstacle to clear interpretation of the impact of the NP-protein interactions on the biological response *in vitro* and *in vivo*.

**Figure 1:**
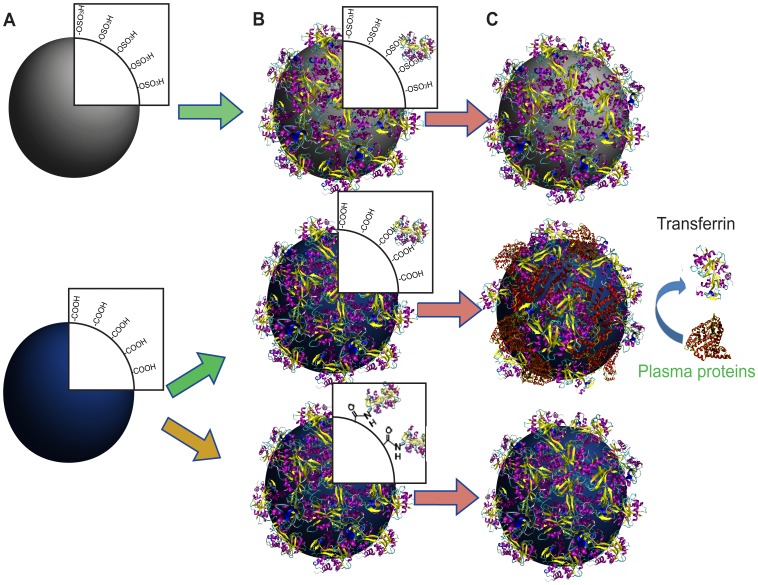
Strategies of PS NPs functionalization with Tf. Cartoon representation of 100 nm polystyrene NPs modified with sulphonated groups (PSOSO_3_H, upper) and carboxylic groups (PSCOOH, down) coated with Tf by either physical adsorption (Tf@PSOSO_3_H and Tf@PSCOOH) or covalent coupling (Tf-PSCOOH): A, pristine NPs; B, Tf coated NPs; C, Tf coated NPs after incubation in human plasma. Both Tf@PSOSO_3_H and Tf-PSCOOH NPs are stable in human plasma, while Tf on Tf@PSCOOH NPs is easily replaced by environmental proteins.

Here, we outline a methodology to address the different aspects governing the formation and the function of the protein corona. In this regard, we functionalize standard polystyrene NPs (bearing either sulphonated or carboxylated groups) with Transferrin (Tf), using two different immobilization strategies and we use these modified NPs to examine the relevant aspects to consider when nanomaterials are intended for a biological use. In particular, we coat the surface of polystyrene NPs, namely sulphonated (PSOSO_3_H) and carboxylated (PSCOOH), with human holo-transferrin (Tf) by both physical adsorption and covalent bonding, and study their stability in human plasma. While PSOSO_3_H NPs are only coated by physical adsorption (Tf@PSOSO_3_H), carboxylic NPs are coated by both physical adsorption and covalent bonding (Tf@PSCOOH and Tf-PSCOOH, respectively) (Figure 1a–b). Holo-Transferrin is an abundant human plasma glycoprotein (about 79 kDa), which transports iron and binds to the Tf-receptor (TfR) in the iron-bound form, activating receptor mediated endocytosis [29], [30]. Since tumour cells have a higher demand for iron they generally overexpress the TfR, thus Tf has been largely used to functionalize nanomaterials, for its potential to target cancer cells [18], [31]–[34]. This study has allowed us to compare Tf-NP bioconjugates for the same core NPs with different functional groups, highlighting their importance in defining the NP affinity to the protein rather than the material of the core. These NPs are characterized in physiological phosphate buffer, to ensure the formation of monodispersed Tf-NP bioconjugates. Then, we investigate their properties in 55% human plasma which allows us to relate any change in the resulting protein-NP complexes to different surface properties and to define NPs bionano surface: protein-NP complexes are studied both *in situ* (in human plasma, full corona FC) and after washing (hard corona, HC) in terms of structural properties, composition and second-order interactions with protein microarrays.

## Results

### Characterization of Tf-coated Nanoparticles

Tf has been passively adsorbed on carboxylated and sulphonated polystyrene nanoparticles (100 nm) to form monodisperse stable Tf coated NPs (Tf@PSOSO_3_H and Tf@PSCOOH). Sulphonated NPs are relatively hydrophobic so the protein binding is expected to be stronger than for carboxylic ones. This is also shown by the estimated amount of Tf adsorbed to NPs surface, which is ∼1.2 times higher for PSOSO_3_H NPs (see Table 1). Carboxylated-modified NPs have also been covalently coupled with Tf resulting in the formation of stable Tf-conjugates. Details about the procedures used to functionalize these NPs are reported in the Experimental Section.

**Table 1:** DLS analysis of bare and Tf coated polystyrene nanoparticles in PBS (pH 7.4) at 25°C.

Sample	D_H_ [nm][a]	PDI[b]	z-potential [mV]	Tf [µg/mgNP][c]
PSOSO_3_H	129.9+/−2.2	0.099	−35.7	–
Tf@PSOSO_3_H	146.4+/−2.1	0.075	−10.4	154+/−9
PSCOOH	117.8+/−2.2	0.034	−38.6	–
Tf@PSCOOH	141.9+/−6.3	0.080	−14.4	125+/−19
PSCOOH-Tf	135.5+/−6.7	0.071	−12.2	165+/−25

[a] z-average hydrodynamic diameter extracted by cumulant analysis of the data.

[b] Polydispersity index from cumulant fitting.

[c] Tf amount for nanoparticle estimated according to the procedure described in method section.

**Figure 2:**
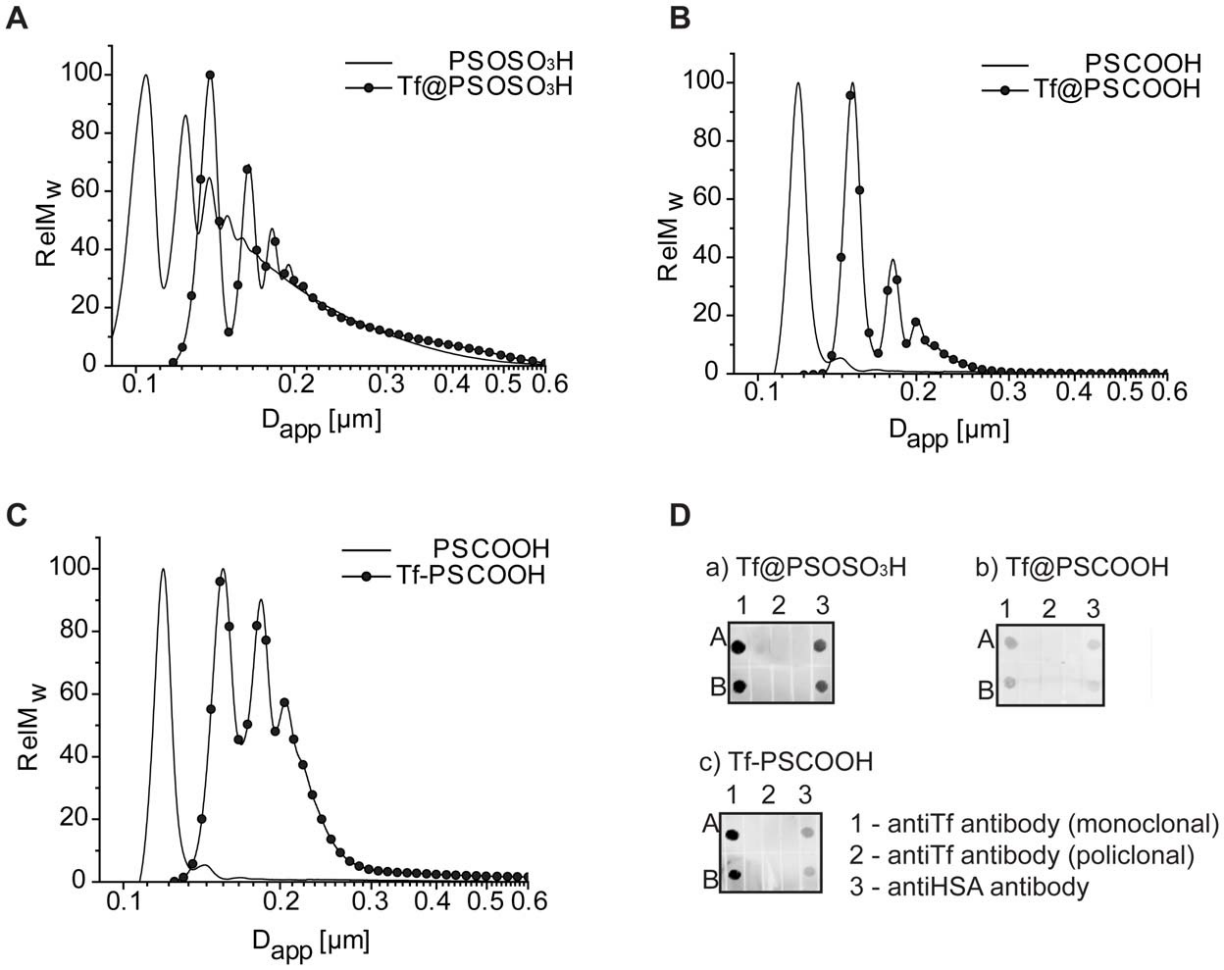
Characterization of Tf coated NPs in PBS at room temperature. Differential centrifugal sedimentation (DCS) results for: A, 100 nm PSOSO_3_H NPs and Tf@PSOSO_3_H NPs; B, 100 nm PSCOOH NPs and Tf@PSCOOH NPs and C, 100 nm PSCOOH NPs and Tf-PSCOOH. All size distributions show a shift towards larger apparent sizes in the presence of Tf coating. D, immuno detection of Tf coated NPs against monoclonal anti-Tf and anti-HAS.

These NPs were characterized by Dynamic Light Scattering (DLS), Z-potential measurements and Differential Centrifugal Sedimentation (DCS), to ensure the formation of monodispersed, and stable bio-coated nano-objects. The increase in the hydrodynamic diameters without major changes in the PDI together with the drop in the z-potential upon Tf conjugation for all samples indicates formation of Tf coated NPs (Table 1). More resolved DCS measurements reveal more details about the size distribution of the bio-conjugates with respect to the unfunctionalized NPs: the particle size distribution resembles that of the pristine NPs for Tf@PSOSO_3_H, while for both Tf@PSCOOH and Tf-PSCOOH NPs, even if the monomer population is always dominant, a tendency to form multiparticle-protein assemblies (mostly dimer and trimer) is observed (Figure 2A–C). The formation of dimer and trimer complexes is more enhanced for covalently coupled Tf-PSCOOH NPs where possible protein cross-linking can occur with further particle bridging. From DCS data, knowing the shape and internal density distribution of the conjugates, it is possible to compute the thickness of Tf-shell for the monomer Tf-NP conjugates. This can be done using a simple core-shell two-density model involving the particle material and the adsorbed protein densities (see Model S1). Tf shells (δ_Tf_) reported in Table 2 are about 4–5 nm for all samples, which is in good agreement with the formation of a full Tf coverage. The structural characterization indicates the formation of stable well-dispersed Tf-coated NPs.

**Table 2:** DCS characteristics of Tf precoated NPs; hard corona (HC) and full corona (FC).

Particle	D_app_ PBS[nm]	δ_Trf_ [nm]	D_app_(HC) 5 min[nm]	δ_HC_ 5 min[nm]	D_app_(HC)1 h [nm]	δ_HC_ 1 h[nm]	D_app_(FC)1 h [nm]	δ_HC_ 1 h[nm]
PSOSO_3_H	104.5	–	agg	–	agg	–	agg	–
PSCOOH	119.2	–	–	–	160.8	∼5.5	175.0	∼7.5
Tf@PSOSO_3_H	138.6	∼4.5	136.9	∼4.0	136.4	∼4.0	143.4	∼5.0
Tf@PSCOOH	151.2	∼4.0	161.9	∼5.5	162.7	∼5.5	172.3	∼7.0
PSCOOH-Tf	155.5	∼4.5	156.0	∼4.5	155.9	∼4.5	155.0	∼4.5

Immunological assay (dot-blots) with monoclonal anti-Tf and anti-HSA gives a strong recognition of functionalized NPs by anti-Tf, as expected, and a weaker recognition by anti-HSA, indicating presence of small amount of albumin in the coating of all samples (Figure 2D). The binding of Tf@NPs at the specific regions of the dot-blots has been revealed by detection of NPs fluorescence. Albumin is also present, in low percentage, in the Tf stock solution (being the most abundant protein in human plasma) and, considering its lower concentration during incubation, the adsorption on the NP surface implies a higher affinity of albumin to associate to the NPs than Tf. MS analysis on the removed protein layer for physically coated NPs indicate that the percentage of albumin is less than 1% and about 1.5% of the total protein amount for Tf@PSOSO_3_H and Tf@PSCOOH, respectively (Table S1 in the SI).

### Protein Corona Structure and Composition of Tf-coated NPs in Human Plasma

**Figure 3:**
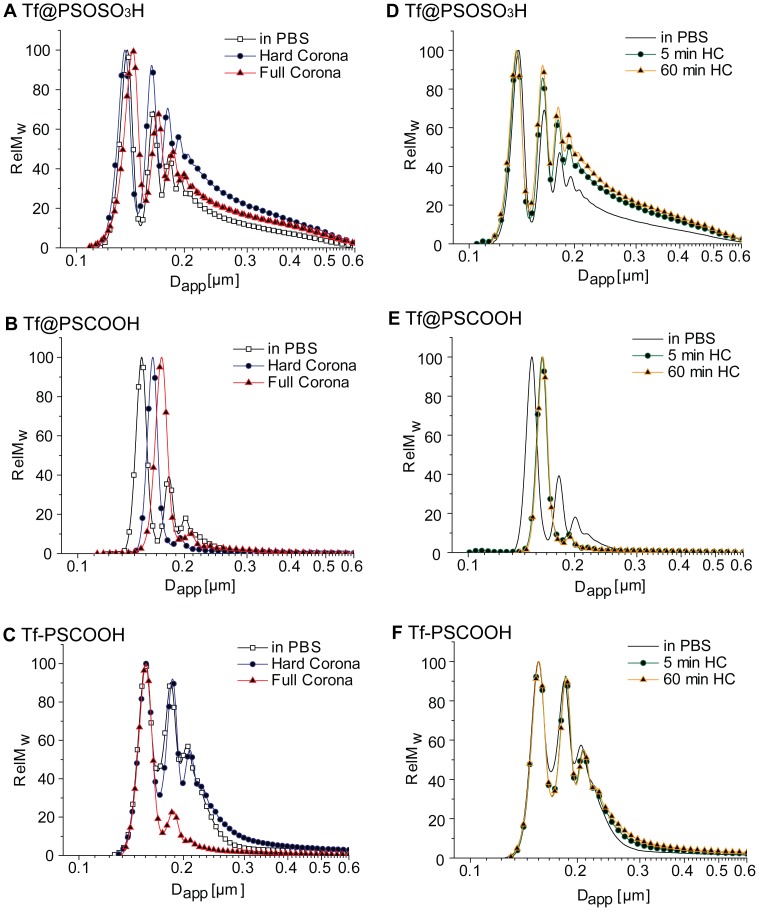
DCS measurements of Tf coated NP-corona complexes before and after incubation in plasma. A–C, apparent size comparison between Tf coated NPs dispersed in PBS, NPs incubated in plasma and isolated by spinning (so called hard corona) and NPs incubated in plasma *“in situ”* (so called full corona); D–F, the particle-corona complexes were isolated after either 5 min or 1 hour of incubation in the biological fluid. DCS experiment for the same NPs in PBS is reported for comparison in all graphs.

We study the behaviour of Tf-coated NPs *in situ* in 55% human plasma solution (v/v in PBS). DCS measurements of NP-protein complexes *in situ* in 55% plasma (full corona, FC) and once isolated from plasma (hard corona, HC), show that these complexes can be physically isolated from the surrounding medium and studied in detail, without significantly altering their structure and size distribution (Figure 3A–C). Moreover, the formation of NP-protein complexes occurs in the first five minutes of incubation (Figures 3D–F). The evaluation of the thickness of the protein corona shell shows that Tf pre-coating inhibits the protein corona formation with respect to the pristine polystyrene NPs [7], [35] for all NPs, but to a different extent (reported in Table 2). Tf@PSOSO_3_H FC complexes *in situ,* although slightly larger, preserve the same size distribution as in PBS, suggesting minor adsorption/exchanging with the environmental proteins. In fact, the corresponding HC complexes isolated from the excess of plasma show the same size as in PBS indicating that the environmental proteins are weakly associated to the NPs (see Figure 3A,D). Tf@PSCOOH FC complexes *in situ* and isolated from plasma (HC) are more monodispersed than in PBS, but show the formation of an extra protein layer on the Tf coating, implying major exchange/adsorption with the environmental proteins (Figure 3B,E). Finally, Tf covalent linkage to the NP surface appears to be more effective in hampering protein adsorption from the environment; in fact both FC and HC NP-protein complexes do not show major changes in the size of monomer complexes with respect to those in PBS (Figure 3C,F).

**Figure 4:**
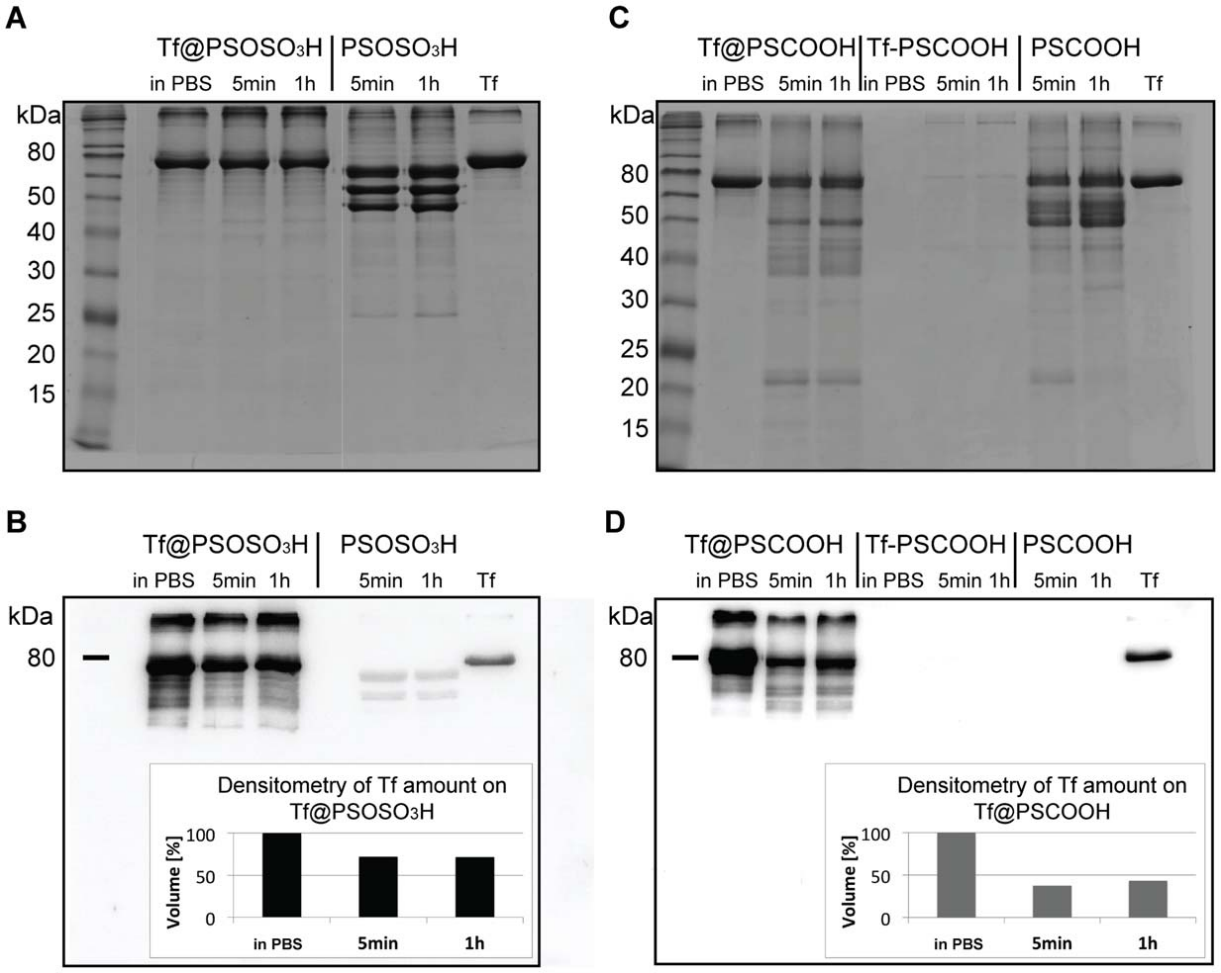
Evaluation of Tf content in the NP coatings by SDS-PAGE and Western Blot. Analysis of the protein coating in PBS and after incubation in 55% human plasma. A, SDS-PAGE of Tf@PSOSO_3_H NPs in PBS after 5 min and 1 hour of incubation in human plasma (after washing). Protein coronas of the pristine PSOSO_3_H NPs after 5 min and 1 hour incubation are reported as reference; B, western blot analysis of the gel reported in Figure A against polyclonal anti-Tf and densitometry of the bands; C, SDS-PAGE of Tf@PSCOOH and Tf-PSCOOH NPs in PBS and after 5 min and 1 hour of incubation in human plasma (after washing). Protein coronas of the pristine PSCOOH NPs after 5 min and 1 hour incubation are reported as reference; D, western analysis of the gel reported in panel C against polyclonal anti-Tf and densitometry of the bands.

Figures 4A and 4C illustrate SDS-PAGE of the protein corona of Tf coated particles in comparison to pristine polystyrene NPs incubated in 55% plasma at different incubation times (5 min and 1 hr). The results are in very good agreement with the structural investigation: Tf@PSOSO_3_H and covalently bound Tf-PSCOOH NPs do not show major adsorption of environmental proteins upon incubation in plasma, while the protein corona composition of Tf@COOH NPs is significantly affected by plasma incubation. The latter sample shows a major decrease in the intensity of the 80 kDa band (Tf molecular weight) and detection of new bands at lower M_w_s. A more quantitative evaluation of the loss of Tf after incubation in human plasma is achieved by WB (Figures 4B and 4D). Western-blot analysis of the protein corona of Tf coated NPs incubated in plasma, against polyclonal anti-Tf antibody shows that Tf exchange with environmental proteins occurs as soon as the particle comes into contact with the fluid, since no changes are detected over 1 hour. In particular, Tf desorption qualitatively evaluated by densitometry analysis of the bands is about 30% and 60% from sulphonated and carboxylated NPs, respectively. The protein-coating detached from Tf-PSCOOH NPs is not recognised by anti-Tf antibody as expected (Figure 4D). In fact, Tf-PSCOOH NPs are extensively purified to remove any excess of unbound Tf after their synthesis (see Figure S1) and SDS-PAGE after incubation in 55% plasma does not show formation of a multi-protein corona (Figure 4C).

MS analysis agrees with these results, showing a large decrease in the detection of Tf upon incubation in plasma for carboxylated Tf coated NPs and a slight diminution for the sulphonated ones (Table 3). MS analysis gives more insights on the composition of the gel band at 80 kDa and indicates histidine-rich glycoprotein and Fibrinogen/HSA as the most abundant proteins for sulphonated and carboxylated Tf@NPs, respectively. MS analysis of the moiety of the gel corresponding to 80 kDa for covalently coated carboxylated NPs highlights the presence of environmental proteins, but the low values of the peptide hits indicate their low abundance.

**Table 3:** Mass spectrometry data for human Tf amount on precoated PS nanoparticles before and after incubation in human plasma.

Sample	Peptide Ions [Hits]	Norm Peak Area [% area]
Tf@PSOSO_3_H	143.00	99.12
Tf@PSOSO_3_H 5 min inc. (55% plasma)	125.00	96.52
PSOSO_3_H 5 min inc. (55% plasma)	24.00	1.21
Tf@PSCOOH	182.00	97.82
Tf@PSCOOH 5 min inc. (55% plasma)	106.00	58.74
PSCOOH-Tf	33.00	58.51
PSCOOH-Tf 5 min inc. (55% plasma)	7.00	3.22
PSCOOH 5 min inc. (55% plasma)	19.00	1.20

### Interactions of Tf-coated NPs at the Bionano Interface

**Figure 5:**
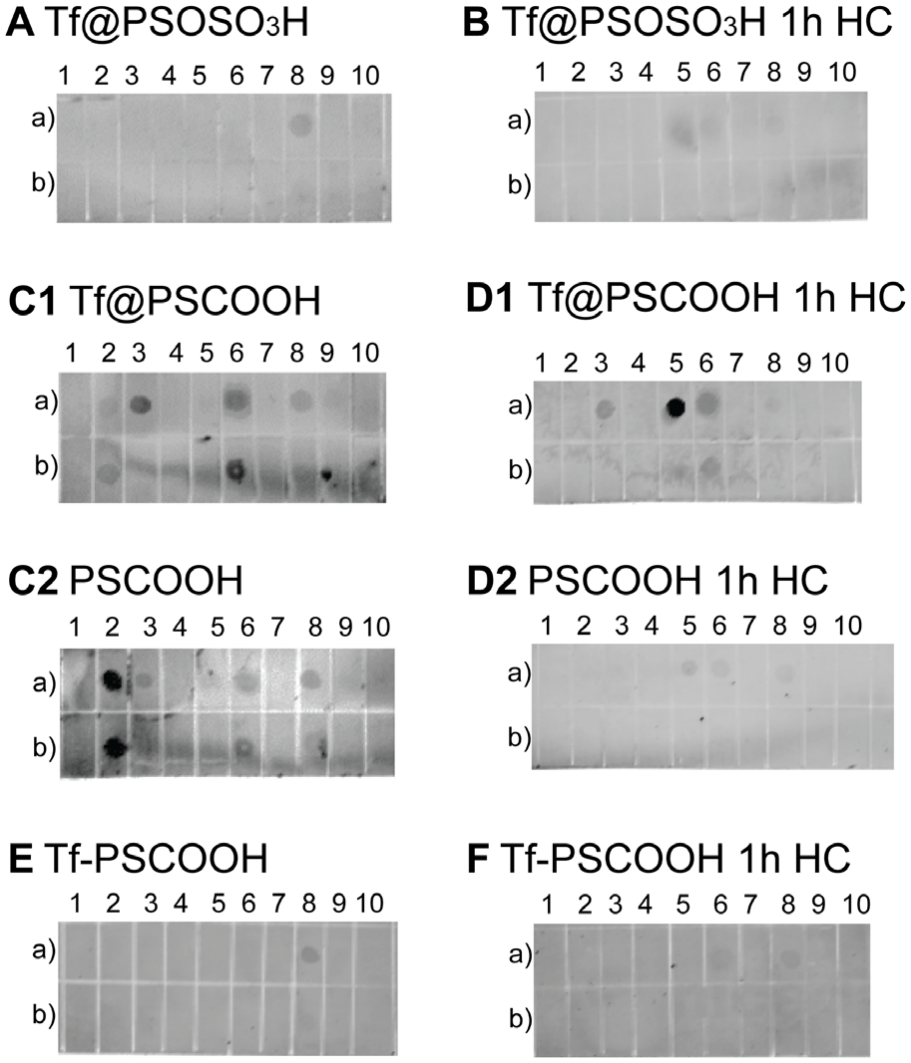
Human plasma protein dot-blots of Tf coated NPs and their HC in 55% plasma. 1 µM (a) and 0.1 µM (b) solutions of human plasma proteins were spotted on the membrane to interact with functionalized NPs. Protein list: 1) HSA 2) Fibrinogen 3) Apo-E 4) Apo-AI 5) Plasminogen 6) Apo-B 7) Apo-AII 8) IgM 9) IgG and 10) Apo-C1.

We have shown that the investigated Tf coated polystyrene NPs are characterized by a different bionano interface in human plasma and presumably this will affect their interaction with the living matter *in vivo*. We further explore the extrinsic physical interactions displayed at the bionano interface formed in the relevant biological fluid studying the binding of protein corona complexes with selected human plasma proteins. Figure 5 qualitatively illustrates that Tf coated NPs in PBS interact differently with the spotted proteins, in particular both Tf@PSOSO_3_H (Figure 5A) and Tf-PSCOOH (Figure 5E) NPs do not show strong interaction with the spotted proteins, giving only a faint signal for IgM. Upon incubation in plasma Tf@PSOSO_3_H (Figure 5B) and Tf-PSCOOH (Figure 5F) HC complexes do not show major changes in the interaction with the spotted proteins with respect to in PBS, confirming the stability of Tf coating. Instead, Tf@PSCOOH NPs (Figure 5C1) give a more complex interaction pattern, which is remarkably similar to the one of the original carboxylated NPs (Figure 5C2). The only difference is that the affinity to Fibrinogen, the strongest hit for the pristine carboxylated NPs, is significantly reduced by Tf coating. In plasma, Tf@PSCOOH HC complexes are again very similar to that of the bare PSCOOH. This behaviour is probably related to the formation of very comparable protein coronas in plasma for these two particles (gel reported in Figure 4C). As expected, the “covalent linkage” approach results in a more stable coating whose extrinsic interactions are not significantly affected by the biological milieu as shown in Figures 5E–F. Direct Tf covalent conjugation to PSCOOH NPs decreases the surface energy of the nano-object, reducing plasma protein adsorption with almost no formation of the protein corona. Thus, the protein coating, although decreasing the surface charge and electrostatic repulsion energy, reduces protein binding on exposure to plasma [36], [37]. However, Tf@PSOSO_3_H NPs do not show significant exchange with environmental proteins and extrinsic interactions seem preserved in the relevant biological milieu. Accordingly, physically adsorbed bio-coatings can be very stable *in situ* in a relevant biological fluid as well, when the interaction between the functional groups on the NP surface and the biomaterial is sufficiently strong.

A better understanding of the resulting interaction pattern (‘interactome’) of Tf@PSOSO_3_H protein corona complexes in human plasma is obtained studying their functional interaction with 9,483 full-length eukaryotically expressed proteins on high content human protein microarrays (Table S2 in the SI). In biological systems, not all of these proteins will be accessible to plasma-borne particles, but they usefully represent the *in situ* biological identity of the particles if compared to their ‘interactome’ in PBS. In fact, this approach could be also used to test the specific biological interactions displayed by Tf coated layers with respect to the free protein, i.e. the ability of Tf to exploit its biological function when adsorbed or linked to the NP surface. It is important to note that neither TfR nor other known functional binders of Tf (STRING algorithm, http://string-db.org/) (see Table S3) are present in these commercially available high content human protein microarrays. Indeed, fluorescently labelled Tf does not show any specific interactions with the arrayed proteins (data not shown).

The main purpose of our present study is to evaluate how physical interactions between Tf@PSOSO_3_H NPs and arrayed human proteins change in the presence of environmental proteins, when different competing processes are simultaneously taking place: protein corona complexes interacting with arrayed proteins, free plasma proteins interacting with protein corona complexes and free proteins interacting with arrayed proteins [11]. However, in the future custom arrays could be used to test the binding of functionalized NPs to selected targets *in situ*, in the biological fluid.

**Figure 6:**
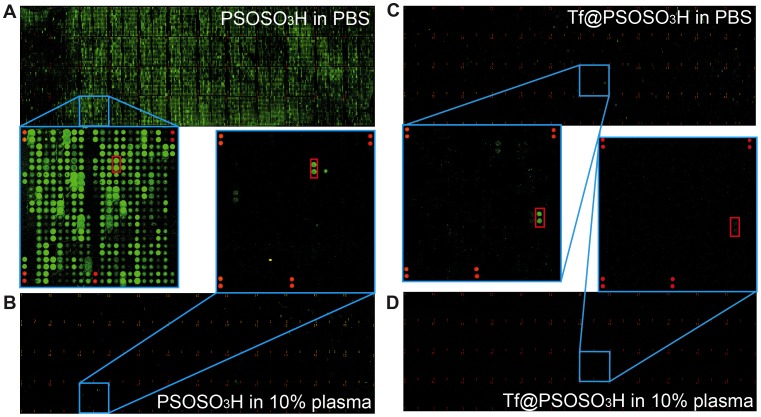
Tf@PSOSO_3_H and PSOSO_3_H NPs incubated on protein arrays. Images of complete protein microarrays incubated with: A, 40 µg/ml PSOSO_3_H NPs in PBS (and sub-array 9); B, 40 µg/ml PSOSO_3_H in 10% human plasma (and miniature of sub-array 9); C, 40 µg/ml Tf@PSOSO_3_H NPs in PBS (and sub- array 26); D, 40 µg/ml Tf@PSOSO_3_H NPs in 10% human plasma (and miniature of sub-array 26).

**Figure 7:**
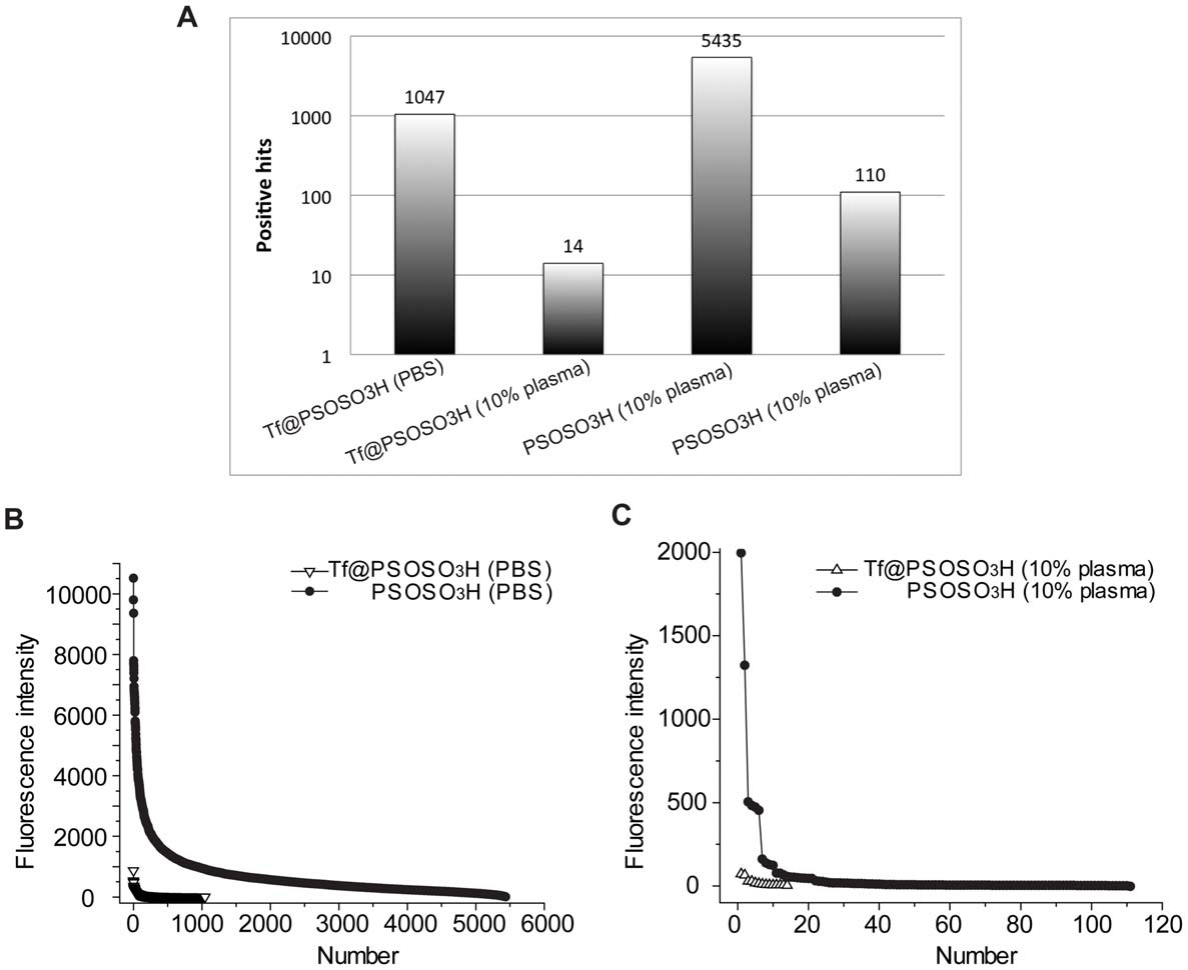
Human protein arrays positive hits comparison. A, graph of total positive hits for bare and functionalized NPs in PBS and 10% human plasma solution; B, fluorescence intensity comparison for positive hits, between bare PSOSO_3_H NPs and Tf@PSOSO_3_H in PBS; C, fluorescence intensity comparison for positive hits, between PSOSO_3_H NPs and Tf@PSOSO_3_H in human plasma.

We investigate four different samples on the protein microarrays: pristine PSOSO_3_H and Tf@PSOSO_3_H NPs both in PBS and in 55% human plasma in the same experimental conditions. It is noteworthy that uncoated NPs form large protein-NP aggregates in plasma as reported in Table 2 and in previous studies [7], thus the corona complexes are very different both in terms of structure and composition from those formed by Tf@PSOSO_3_H. We find that sulphonated NPs, unsurprisingly (due to their high surface energy), in the absence of plasma bind to most proteins on the array (Figure 6A) where 5500 positive spots can be detected, and the majority of these interactions are similar to each other (Figures 7A–B) indicating that non-specific interactions between particle surface and typical proteins are similar across the array. The strength of all these bare surface-protein interactions strongly decreases over incubation in human plasma, when protein-NP complexes are formed (see Figure 6B). In this case, the total number of positive spots lowers to about 100 (see Figure 7A,C), but most of the interactions are comparable in term of strength (on the base of fluorescent intensity), presumably revealing a typical energy scale of non-specific interactions between hard corona and target array proteins. The most intense positive spots are from proteins, which cannot be related to any known specific interaction with the proteins in the corona of the NPs.

Tf coating on sulphonated NPs, as expected, strongly decreases the strength of interactions with the arrayed proteins both in terms of intensity and number of positive spots, but the general trend of the binding profile (Figure 7B), which resembles that of pristine sulphonated NPs, reflects a non-specific nature of most interactions. A more detailed analysis reveals that most of the proteins in the array bound by Tf@PSOSO_3_H NPs are positive for the pristine sulphonated NPs as well, with the only exception of dynamin 2, the second highest positive spot (Tables S4a and S4c in the SI), which does not present any known relation to Tf. Finally, the “interactome” of Tf@PSOSO_3_H NPs in human plasma, where there is no evidence of the formation of a thick robustly bound protein corona, is strongly decreased in number and strength of the interactions (Figure 6D and 6C), indicating that the competition with free environmental proteins is significantly affecting the binding pattern to the arrayed proteins. It is also interesting that among the 14 interactions only five are shared with those displayed in PBS (see Table 4), which appear more intense in absence of plasma. The 10 emergent new interactions in the biological fluid are not listed as known binders to either Tf or to other plasma proteins.

**Table 4:** The fluorescence intensity for positive interaction hits for Tf@PSOSO_3_H in 10%plasma and PBS.

No	Protein name	10% plasma	PBS
1	Homo sapiens hypothetical protein DKFZp434E2220 (DKFZp434E2220), mRNA	72.5	365.5
2	Homo sapiens outer dense fiber of sperm tails 2, mRNA (cDNA clone MGC:9034 IMAGE:3874501), complete cds	65	–
3	Homo sapiens calcium binding and coiled-coil domain 1 (CALCOCO1), mRNA	29	–
4	Homo sapiens complexin 1 (CPLX1), mRNA	28.5	37.5
5	Homo sapiens centaurin, delta 2 (CENTD2), transcript variant 1, mRNA	18	38.5
6	Homo sapiens TruB pseudouridine (psi) synthase homolog 1 (E. coli) (TRUB1), mRNA	15	104
7	Homo sapiens mitochondrial ribosomal protein S23 (MRPS23), nuclear gene encoding mitochondrial protein, mRNA	11.5	–
8	Homo sapiens mitogen-activated protein kinase 9 (MAPK9), transcript variant JNK2-a2, mRNA	8.5	–
9	Removed from database	7	–
10	Homo sapiens protein kinase, AMP-activated, gamma 2 non-catalytic subunit (PRKAG2), mRNA	6	117
11	Homo sapiens mRNA similar to LOC166173 (cDNA clone IMAGE:5203730)	6	–
12	Homo sapiens paraoxonase 3, mRNA (cDNA clone MGC:88384 IMAGE:4710338), complete cds	6	–
13	Homo sapiens lactate dehydrogenase A (LDHA), mRNA	6	–
14	Homo sapiens lipopolysaccharide-induced TNF factor (LITAF), mRNA	4	–

## Discussion

The generally accepted view in nanomaterial research for biological applications is that the biologically relevant unit is not the pristine particle, but a nano-composite of specified size, shape, and selective protein corona structure. It has been extensively elucidated how NP size and functional groups can significantly shape the protein corona of the same bulk material nanoparticles [12], [16], [38], [39] and here we address the effect of a protein coating on the formation of the corona in a biological fluid. Our results clearly show how functional groups determine the stability of physically adsorbed protein-NP conjugates much more than the bulk material. In fact, if NP incubation with Tf succeeds in the formation of stable, monodisperse monomer Tf coated conjugates, for both sulphonated and carboxylated polystyrene NPs (see Figure 2 and Table 1), DCS results on FC and HC protein complexes clearly indicate that physical Tf pre-coating inhibits the formation of a robust protein corona in the biological environment for sulphonated NPs (see Table 2 and Figure 3), but not for carboxylated ones. These structural findings are strongly supported by the biological investigation (SDS-PAGE, WB and MS analysis) reflecting very little exchange of Tf with the environmental proteins for Tf@PSOSO_3_H NPs. Tf@PSCOOH NPs, instead, exhibit a rearrangement at the bionano interface with a big loss of Tf and consequent formation of a multi-protein corona after instantaneous contact with environmental proteins. The composition of this protein corona, although Tf-enriched, resembles the protein corona of pristine carboxylated NPs. As expected, Tf covalent linkage to the surface of carboxylated NPs provides a stable coating with the formation of monomer bio-conjugates whose structure and composition is almost invariant on exposure to plasma (Figure 4C). This does not guarantee the preservation of the native conformation of the grafted protein (random coupling between amine groups of the protein with carboxylic groups on the NP’s surface) and of its biological function. An additional study on how the covalent coupling strategy used to modify nano-scaled surfaces with protein (grafting density, coupling reaction, presence of linker, etc.) affects their behaviour in a relevant biological fluid and their specific binding to the biological target is currently in progress in our laboratory.

In order to elucidate the extrinsic interactions of these nanoparticles *in situ*, we model the interaction of proteins resident in the corona of the nanocomposite on high content protein microarrays. First, we use human plasma protein dot-blots (Figure 5), as a simple method to probe the interaction of the new bionano interfaces with the environmental proteins with respect to the pristine NPs. For example, the evidence that Tf@PSCOOH and bare PSCOOH HC complexes give similar binding pattern with human plasma spotted proteins (Figure 5D1 and 4D2), suggest that preserved Tf (after exposure to plasma) is strongly associated to the NP surface and does not seem to affect the physical interactions displayed by these nano-composites *in situ.* This result shows how such a simple assay as a dot-blot can give important information to better outline the local structure of the protein corona. This result also indicates that the preserved Tf on the NP surface will very likely be unable to interact with the desired bio-target (TfR) *in vivo*. For this reason the more sophisticated experiments with microarrays were only made with the Tf@PSOSO_3_H NPs.

The biological environment is extremely complex and it is difficult to reproduce it *in vitro*, thus we propose the use of high content human protein microarrays as a new approach to dissect the “interactome” of these NPs *in situ.* General outcomes from these experiments are the following, although the repertoire of the proteins spotted on arrays is not complete. Lower surface energy Tf coated NPs, as expected, show lower tendency to bind to the arrayed proteins than bare sulphonated NPs, and the binding profile does not reflect any emergent specific interaction except to one protein, dynamin 2, with respect to uncoated sulphonated NPs in PBS. The possibility of a new specific interaction between adsorbed Tf and dynamin, due to the possible exposition of new binding sites for Tf upon adsorption on the NP surface is unlikely, as this interaction is not further detected when the NPs are incubated with the microarray in human plasma. The significant decrease in number and strength of positive spots for pristine sulphonated NPs in human plasma is also expected, as environmental proteins compete in the binding and protein-NP complexes will have a lower affinity to the arrayed proteins (lower surface energy). Moreover, even if specific interactions between protein resident in the corona and arrayed proteins are not detected, one should be aware of potential exposure of new binding sites (not necessarily specific) giving new unknown interactions. In this case, any of the emerging proteins that interact with the protein corona NPs is relevant to their interaction with the living matter. However, the most surprising outcome is the abrupt change in the binding pattern of Tf@PSOSO_3_H NPs to the arrayed proteins in plasma with only 14 significant hits. As hard corona complexes seem to preserve the original structure and composition of the original Tf coated NPs, this change cannot be simply related to the formation of a hard corona layer, unless we involve a contribution of the soft corona, i.e. the loosely bound proteins in fast exchange with the environment. In fact, we believe that this result indirectly reflects a very significant role of the competitive binding displayed by free environmental proteins in the interaction between protein corona NPs and living matter (in this case represented by the arrayed proteins). Presumably, many binding sites are now engaged and not available to interact with Tf coated NPs, which are less prone to give physical non-specific interactions than multi-protein corona complexes as in the case of the pristine sulphonated NP-corona complexes (Figure 6B,D and Figure 7A). This can be explained by the presence of more “binding sites” on the multi-protein corona complexes, due to their richer protein composition. Moreover, none of the 14 positive spots are among the brightest spots in PBS, suggesting that those interactions (in PBS) are not specific and easily switched off by stronger binders present in the medium. Only five of these proteins are also bound by Tf@PSOSO_3_H in PBS (not the top five) indicating that the competitive binding from free medium proteins can also induce a change in the interaction profile with certain proteins.

Here, we have functionalized different polystyrene NPs (carboxylated and sulphonated) with Transferrin by both physical adsorption and covalent bonding, as a relevant example to address the important aspects governing the behaviour of bio-coated NPs *in situ* in a biological fluid. We present an integrated methodology where we show the importance to combine physico-chemical, biological and proteomic techniques for extensive characterization of the properties and the interactions of these NPs *in situ*. We show that the surface chemistry of the NPs has the major impact on the strength of the interaction with Tf itself as well as on the further stability of the adsorbed coating in human plasma: both structural (DCS) and composition analysis (SDS-PAGE, WB and MS) of the protein corona indicate that Tf@PSOSO_3_H and Tf-PSCOOH NPs are stable and do not form strong protein-NP complexes. For Tf@PSCOOH NPs, instead, the bionano interface is subjected to rearrangement upon incubation in human plasma with remarkable similarities to that of the carboxylated original NPs, as confirmed by the same binding affinity to the spotted plasma proteins. Finally, we study the interaction of selected NPs with eukaryotic protein arrays in PBS and in human plasma. The number and intensity of the positive spots for Tf coated sulphonated NPs decrease significantly with respect to those for the protein-corona PSOSO_3_H NPs, showing that the absence of a multi-protein corona has a big impact on the extrinsic interactions of the NPs *in situ*. These results are encouraging as they show that, when the Tf coating is strong and stable in the biological fluid (with no formation of a rich protein corona), the affinity to bind arrayed proteins significantly decreases. Presumably, Tf coated NPs will have a chance to compete with environmental proteins in binding the arrayed targets only when a specific interaction is involved, while uncoated polystyrene NPs decorated by a multi-protein corona in plasma show a higher tendency to bind the arrayed proteins. The methodology outlined here could be of some importance in future, for applications whenever engineered NPs are meant to be used in a biological environment to either find out new ligands, due to the exposition of unknown epitopes upon protein absorption on the NP surface, or to test the specific binding to a bio-target. We also stress some limitations of this technology at present, for example the limited repertoire of proteins on the commercially available arrays reducing the number of specific targets. We envisage that the next important step in the use of this technology for studying NP-biomolecule interactions in a biological relevant fluid is to make custom arrays where specific binders and ubiquitous proteins are simultaneously present.

## Materials and Methods

### Materials

Holo-Transferrin Human (cat. no. T4132), HSA (cat. No. A9511), IgG (cat. no. I2511), PBS tablets (cat. no. 3671), MES (cat. no. M3671), EDAC (cat. no. E6383) and glycine (cat. no. G7126) were all purchased from Sigma-Aldrich. Micro BCA Protein Assay Kit and Pierce ECL Western Blotting Substrate (cat. no. 32209) were purchased from Thermo Scientific. Mouse monoclonal anti-Tf antibody (ab769), rabbit polyclonal anti-Tf antibody (ab66952), mouse monoclonal anti-HSA antibody (ab10241) and goat secondary anti-rabbit antibody (ab6721) were purchased from ABCAM. ApoA-I (cat. no.178452), ApoC-I (cat.no. 178459), ApoA-II (cat.no. 178455), Fibrinogen (cat.no. 341576), IgM (cat.no. 401799), ApoE (cat.no. 178468), ApoB (cat.no. 178456) were all purchased from CALBIOCHEM. Transferrin – alexa fluorophore conjugates (T13342 and T35352) were purchased from Invitrogen. The 300 kDa cellulose ester dialysis tubing (cat. no. 131450) was purchased from SpectrumLabs. Blue Loading Buffer for SDS-PAGE was ordered from New England Bio-Labs (cat. no. B7703S).

### Nanoparticles

Fluorescent Polystyrene latex beads, both carboxyl-modified PSCOOH (100 nm) and sulphonated-modified PSOSO_3_H (100 nm), were purchased from Invitrogen by Life Technologies. All nanoparticles were characterized by measuring their size and z-potential in physiological buffer before use.

### Human Plasma

Blood was withdrawn from 10–15 different volunteers and collected into 10 ml K_2_EDTA coated tubes (BD Bioscience). Plasma was prepared following the HUPO BBB SOP guidelines [40]. Briefly, immediately after blood collection, each tube was inverted ten times to ensure mixing of blood with the EDTA, and subsequently centrifuged for ten minutes at 1300 g at 4°C. Equal volumes of plasma from each donor were collected into a secondary 50 ml falcon tube and then centrifuged at 2400 g for 15 minutes at 4°C. Supernatant was collected (leaving approximately 10% of the volume in the secondary tube) and it was then aliquoted into 1 ml cryovials and stored at −80°C until use. The whole procedure did not take more than three hours. Following this procedure the plasma protein concentration is estimated to be ∼80 g/L. When plasma was used for experiments, it was allowed to thaw at room temperature and centrifuged for 3 min at 16.2 kRCF. Thawed plasma was never re-frozen or re-thawed. All data presented are obtained using plasma from one donation session. The blood donation procedure was approved by the Human Research Ethics committee at University College Dublin.

### Preparation of Physically Adsorbed Tf Nanoparticles (Tf@PSCOOH, Tf@PSOSO_3_H)

Physically adsorbed Tf@NPs were prepared by incubating bare polystyrene carboxylic-modified and sulphonate-modified 100 nm NPs in Tf solution in 50 mM MES (2-(*N*-morpholino)ethanesulfonic acid) buffer at pH = 5.9 for two hours at room temperature with further extensive dialysis against 10 mM PBS buffer at pH = 7.4. Tf concentration was optimized preparing a series of samples with constant NP concentration (2.2 mg/ml) and multiple Tf concentrations varying from 0.56 mg/ml to 4.45 mg/ml (see Figure S2). The amount of adsorbed Tf was quantified with use of protein assay (Pierce Micro BCA Protein Assay Kit) after dialysis against PBS buffer. The optimal protein concentration for the adsorption, guarantying full surface coverage was found to be 2.2 mg/ml.

### Preparation of Covalently Bound Tf Nanoparticles (Tf-PSCOOH)

5 mg of Tf was dissolved in MES 50 mM pH = 6 at a concentration of 2 mg/ml. PSCOOH NPs were dispersed in the same buffer at a concentration of 2 mg/ml. Equal volumes of each were mixed by adding particle solution drop-wise to the protein solution with intermittent shaking. The clear mixture was then left to mix in a lab incubator for 10 minutes shaking at 500 rpm, after which 5 mg of EDAC dissolved in 20 µl buffer was added. The mixture was shaken for a further 2 hours before 10 mg of glycine was added to quench the reaction. The dispersion was then dialyzed against 10 mM phosphate buffer pH 7.4 (1×24 hrs) in 300 kDa cut-off membrane followed by dialysis against PBS (3×24 hrs).

### Sample Preparation

Hard protein coronas on both bare and Tf pre-coated NPs were prepared by incubating the NPs at concentration of 0.4 mg/ml in 55% human plasma solution (total protein content 34–47 mg/ml) at room temperature for either 5 minutes or 1 hour. After the incubation, samples were spun down and washed three times with 0.5 ml PBS. The three washing steps allowed for removal of proteins loosely bound to NPs surface (so-called soft corona) with recovering of the so-called hard corona composed of the most affine proteins. The final pellet was re-suspended in varying volumes of PBS depending on the experiment. NP samples *in situ* for DCS experiments were prepared in the same conditions than those specified above for hard corona (without spinning down and washing) and diluted to concentration 0.1 mg/ml with PBS directly prior injection to DCS gradient.

### DLS and Z-potential

DLS measurements at the scattering angle θ = 173 and Z-potential determination were performed using a Malvern Zetasizer 3000HSa. Each measurement was an average of twelve repetitions of ten seconds each and repeated five times. Data analysis has been performed according to standard procedures, and interpreted through a cumulant expansion of the field autocorrelation function to the second order.

### Differential Centrifugal Sedimentation (DCS)

Differential centrifugal sedimentation experiments were performed with a CPS Disc Centrifuge DC24000. The CPS Disc Centrifuge is a particle size analyser for measuring particles in the range of 0.01 µm to 40 µm. The analyser measures particle size distributions using centrifugal sedimentation within an optically clear spinning disc filled with fluid. Sedimentation is stabilized by a density gradient within the fluid, and accuracy of the measured sizes is insured through the use of a known size calibration standard run immediately before each measurement. The use of a biological sample with a large amount of proteins requires a new sucrose gradient to be prepared for each measurement. The concentration of the particles at each size is determined by continuously measuring the turbidity of the fluid near the outside edge of the rotating disc. The turbidity measurements are converted to a weight distribution by Mie theory light scattering calculations. The choice of the experimental parameters, namely the fluid gradient, the speed of rotation, etc. is based on the type of material being analyzed and the range of sizes being measured. The primary information from the analytical disk centrifuge is the time taken for the particles to travel from the centre of the disk through a defined viscous sucrose gradient to a detector placed at the outer rim of the disk under a strong centrifugal force. For materials with homogenous density and simple shape (for example spherical particles) one can directly relate this time to a particle size. Where objects are inhomogeneous, or irregular in shape, the different arrival times still allows one to distinguish between them. In the simplest approximation one still approximates them to an equivalent uniform sphere, and this is the meaning of the size cited on the x-axis of all figures presented and hence it should be considered as an ‘apparent’ size. Moreover, for the sake of clarity in the comparison of different samples, we chose to show data as relative weight particle size distribution. The tallest peak (highest weight value) in the distribution is called the ‘base’ peak (has a value of 100%) and all other particle size (multimer) peaks are then normalized against this base peak to give a relative weight distribution. It is important to emphasize that the conversion from absorption raw data to molecular weight data is correct as long as the optical parameters and the density for particles and fluid are correct and the particles are spherical.

### 1D-SDS-PAGE and WB

Tf@NPs and their hard corona complexes were separated and denatured by boiling for 5 minutes in blue loading buffer (62.5 mM Tris-HCl (pH 6.8 @ 25°C), 2% (w/v) SDS, 10% glycerol, 0.01% (w/v) bromophenol blue, 40 mM DTT). So prepared samples, containing denatured corona proteins coated with SDS surfactant (which gives them negative net charge) were separated by size in the moiety of porous 10% polyacrylamide gel (1D SDS-PAGE), in electric field using a Mini-PROTEAN Tetra electrophoresis system from Bio-Rad. The electrophoresis was run under constant voltage of 130 V for about 45 minutes. All the gels were run in duplicates – one subjected to WB and the second stained with commassie blue (50% methanol, 10% acetic acid, 2.5% (w/v) brilliant blue) for 3 hours and de-stained overnight in 50% methanol, and 10% acetic acid.

For the Western Blots the protein coronas separated by SDS-PAGE were transferred from the gel to PVDF membranes using Mini-PROTEAN Tetra Trans-Blot Module under a constant voltage of 100 V for 1 hour. The membranes were then incubated at RT for 1 h in “blocking solution” of 5% skimmed milk in TBS-TWEEN (150 mM NaCl, 10 mM Tris HCl, 0.1% Tween, pH7.5). Afterwards, blots were incubated (4°C, overnight) with 0.4 µg/ml rabbit polyclonal antibody against human transferrin in “blocking solution” and subsequently washed in 3×15 minutes in TBS-TWEEN. After washing membranes were incubated with 0.1 µg/ml of horseradish-coupled goat anti-rabbit antibody in “blocking solution” for 1 hour in RT and washed 3×15 minutes in TBS-TWEEN and 1×5 minutes in Millipore water. Specific antibody binding was visualized using Pierce Enhanced ChemiLuminescence (ECL) Western Blotting Substrate and X-ray photographic films.

Both Commassie stained gels and Western Blots were scanned using Bio-Rad GS-800 Calibrated Densitometer and densitometry was performed using TotalLab Quant software.

### Mass Spectrometry

After the SDS-PAGE separation of the protein-NP complexes, gel lanes were excised and the bands taken from each lane prior to trypsin digestion and mass spectrometry. All samples were run on a Thermo Scientific LTQ ORBITRAP XL mass spectrometer connected to an Exigent NANO LC.1DPLUS chromatography system incorporating an auto-sampler. Tryptic peptides were resuspended in 0.1% formic acid. Each sample was loaded onto a Biobasic C18 Picofrit™ column (100 mm length, 75 µm ID) and was separated by an increasing acetonitrile gradient, using a 120 min reverse phase gradient (0–40% acetonitrile for 90 min) at a flow rate of 30 nL min^−1^. The mass spectrometer was operated in positive ion mode with a capillary temperature of 200°C, a capillary voltage of 46 V, a tube lens voltage of 140 V and with a potential of 1800 V applied to the frit. All data was acquired with the mass spectrometer operating in automatic data dependent switching mode. A high resolution MS scan was performed using the Orbitrap to select the 5 most intense ions prior to MS/MS analysis using the Ion trap. The raw mass spectral data was analysed using Bioworks Browser 3.3.1 SP1, a proteomics analysis platform. All MS/MS spectra were sequence database searched using the algorithm TurboSEQUEST. The MS/MS spectra were searched against a IPI 3.5 database. The following search parameters were used: precursor-ion mass tolerance of 2 Da, fragment ion tolerance of 1.0 Da with methionine oxidation and cysteine carboxyamidomethylation specified as differential modifications and a maximum of 2 missed cleavage sites allowed.

### Protein Microarrays

The Protoarray microarray slide (Invitrogen) was blocked by incubation in 1% skim milk powder dissolved in PBS with 1 mM DTT, 0.1% Tween 20 and 50% glycerol pH 7.5 for one hour and then washed once in PBS pH 7.5. The 100 nm PSOSO_3_H and Tf@PSOSO_3_H NPs were diluted to a final concentration of 40 µg/ml either in PBS or in a buffer containing human plasma diluted to a final concentration of 10%. Before imaging the slide was washed once in PBS and rinsed in deionized water. Microarray slides were scanned in a Genepix 4000B scanner (Axon Instruments). The PMT gain settings were maintained at 500 and 250 for the 635 nm and 532 nm lasers respectively. The focus position was 10 µm. The microarrays used were all from the same lot and the gal file specific to the lot of microarrays was downloaded from the Invitrogen webpage. The gpr results files from the array scans were analyzed with the Prospector software (Invitrogen) using the small molecule fluorescence settings.

### Dot-blots

The dot-blots were prepared by spotting 1 µL of either the 10 selected plasma proteins (1 µM and 0.1 µM solutions in 10 mM PBS) or selected antibodies (100 µg/ml, 20 µg/ml and 10 µg/ml solutions in 10 mM PBS) on PVDF membrane, previously activated in methanol and equilibrated in 10 mM PBS. So prepared blots were blocked in 5% skimmed milk solution in 10 mM PBS for 1 hour in RT, washed three times for 5 min in 10 mM PBS and incubated in 40 µg/ml NPs solution in PBS for 1 hour at RT. After subsequent 3×5 min washes in PBS, blots were dried and imaged for fluorescence using the “TYPHOON 9200” imager. NPs fluorescence indicate the regions of the blot, to which NPs attached as a result of positive interactions between Tf on NP surface and protein spotted on the dot-blot. Scanner settings: excitation wavelength 532 nm, emission wavelength 555 nm, normal sensitivity, PMT 350 V.

## Supporting Information

Figure S1 **Covalently bound Tf-PSCOOH NPs undergone to dialysis for different times.** The NPs have been treated to strip the Tf layer off from the surface and the recovered protein solution analysed by SDS-PAGE (silver staining). The physical adsorbed Tf component seems to be removed after 98 hours of dialysis.
(TIFF)

Figure S2 **The transferrin adsorption curves for the PSCOOH and PSOSO_3_H NPs**. The increase of the Tf solution concentration while adsorption on NPs leads to increase of the amount of Tf shell on NPs.
(TIFF)

Table S1 **Mass Spectrometry data for Tf coated nanoparticles before and after plasma incubation (55%).**
(DOCX)

Table S2 **ProtoArray® v4.1 content and distribution by class.**
(DOCX)

Table S3 **The main Tf binders according to STRING algorithm.**
(DOCX)

Table S4 **Complete list of positive protein microarray interaction hits for Tf@PSOSO_3_H NPs in PBS (a), Tf@PSOSO_3_H NPs in 10% plasma (b), PSOSO_3_H NPs in PBS (c) and PSOSO_3_H NPs in 10% plasma (d).**
(XLSX)

Model S1 **Core-shell model analysis for DCS data.**
(DOCX)
