# Supplementary figures and images for: Transferrin Coated Nanoparticles: Study of the Bionano Interface in Human Plasma
Source: PLoS One. 2012 Jul 19;7(7):e40685. doi: 10.1371/journal.pone.0040685 (PMC3400652; doi:10.1371/journal.pone.0040685)

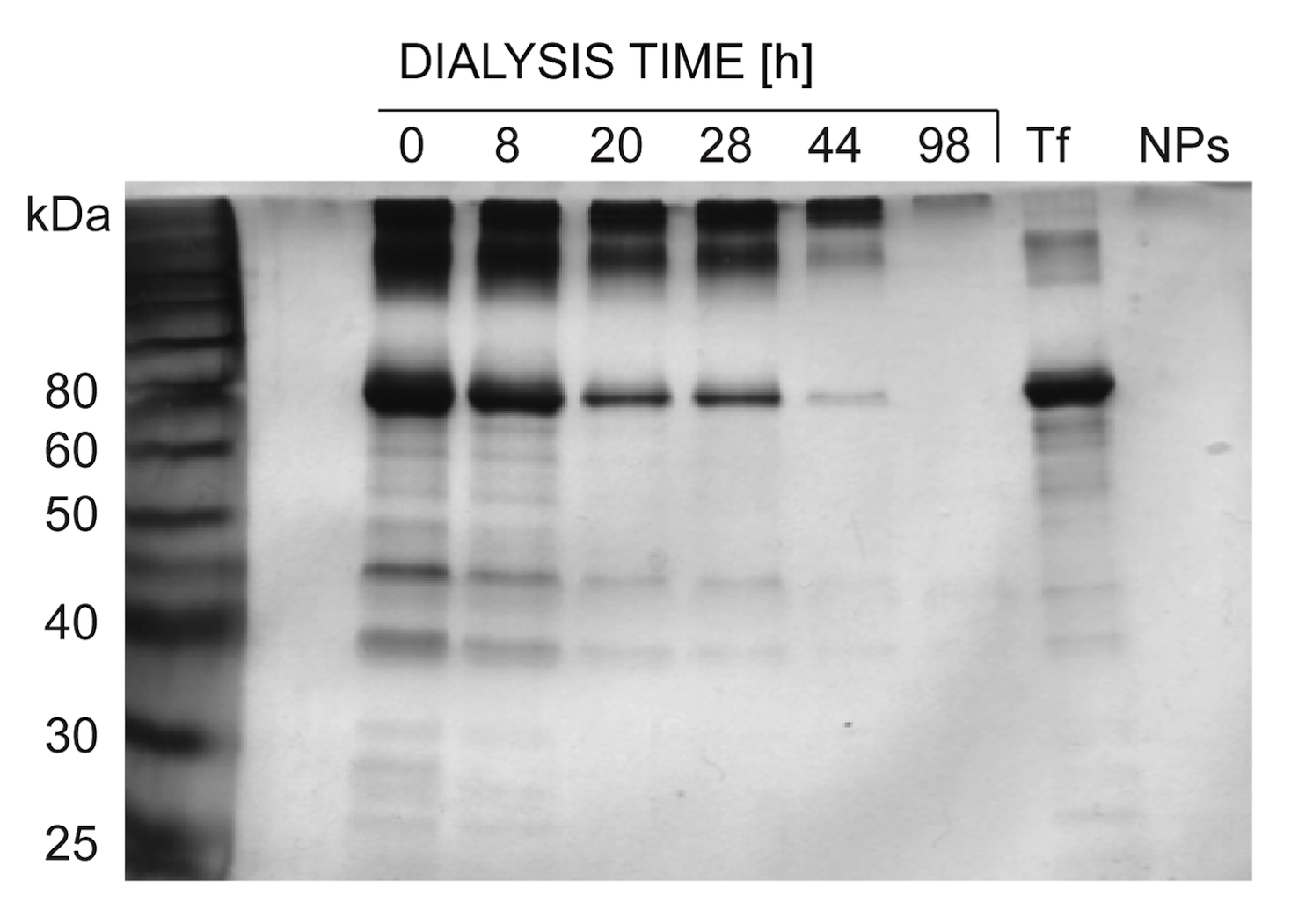

Supplement: Figure S1 — Covalently bound Tf-PSCOOH NPs undergone to dialysis for different times. The NPs have been treated to strip the Tf layer off from the surface and the recovered protein solution analysed by SDS-PAGE (silver staining). The physical adsorbed Tf component seems to be removed after 98 hours of dialysis. (TIFF) [file pone.0040685.s001.tiff]

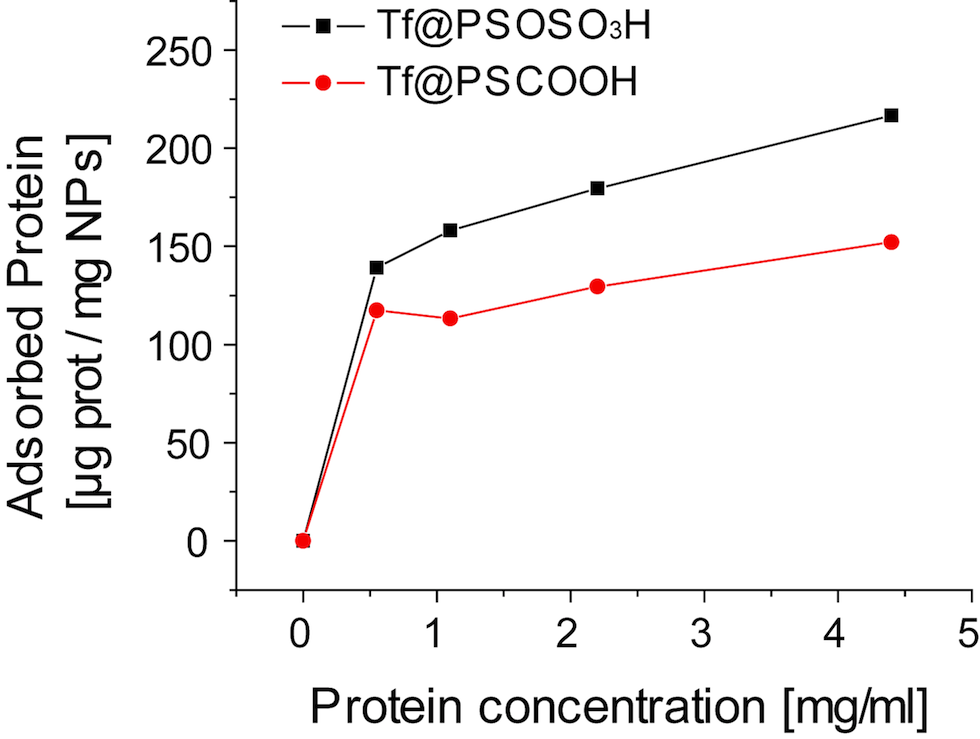

Supplement: Figure S2 — The transferrin adsorption curves for the PSCOOH and PSOSO3H NPs. The increase of the Tf solution concentration while adsorption on NPs leads to increase of the amount of Tf shell on NPs. (TIFF) [file pone.0040685.s002.tiff]
