# Supplementary material for: Transferrin Coated Nanoparticles: Study of the Bionano Interface in Human Plasma
Source: PLoS One. 2012 Jul 19;7(7):e40685. doi: 10.1371/journal.pone.0040685 (PMC3400652; doi:10.1371/journal.pone.0040685)
Supplement: Table S1 — Mass Spectrometry data for Tf coated nanoparticles before and after plasma incubation (55%). (DOCX) [file pone.0040685.s003.docx]

| Tf@PSOSO_3_H | | | | |
| --- | --- | --- | --- | --- |
| **Reference** | **Protein** | **MW** | **Peptide [Hits]** | **Norm Peak Area [%]** |
|  |  | **[Da]** |  |  |
| sp\|P02787\|TRFE | Serotransferrin | 76999.7 | 143 | 99.26 |
| sp\|P02768\| ALBU | Serum albumin | 69321.6 | 23 | 0.74 |
|  | | **sum** | **160** | **100** |
| Tf@PSOSO_3_H  **5min incubation in 55% plasma** | | | | |
| **Reference** | **Protein** | **MW [Da]** | **Peptide [Hits]** | **Norm Peak Area [%]** |
| sp\|P02787\| TRFE | Serotransferrin | 77013.67 | 125 | 97.36 |
| sp\|P02768\| ALBU | Serum albumin | 69321.63 | 26 | 2.04 |
| sp\|P02679\|FIBG | Fibrinogen gamma chain | 51478.88 | 3 | 0.61 |
|  | | **sum** | **154** | **100** |
| PSOSO_3_H  **5min incubation in 55% plasma** | | | | |
| **Reference** | **Protein** | **MW [Da]** | **Peptide [Hits]** | **Norm Peak Area [%]** |
| sp\|P02671\|FIBA | Fibrinogen alpha chain | 94914.27 | 83 | 76.34 |
| sp\|P02768\|ALBU | Serum albumin | 69321.63 | 56 | 14.4 |
| sp\|P02675\|FIBB | Fibrinogen beta chain | 55892.23 | 33 | 2.99 |
| sp\|P02679\|FIBG | Fibrinogen gamma chain | 51478.88 | 15 | 2.8 |
| sp\|P01871\|IGHM | Ig mu chain C region | 49275.57 | 8 | 1.21 |
| sp\|P01024\|CO3 | Complement C3 | 187029.3 | 16 | 0.88 |
| sp\|P04003\|C4BPA | C4b-binding protein alpha chain | 66989.44 | 9 | 0.77 |
| sp\|P06396\|GELS | Gelsolin | 85644.25 | 18 | 0.61 |
|  | | **sum** | **238** | **100** |
| Tf@PSCOOH | | | | |
| **Reference** | **Protein** | **MW[Da]** | **Peptide [Hits]** | **Norm Peak Area [%]** |
| sp\|P02787\|TRFE | Serotransferrin | 76999.7 | 182 | 98.47 |
| sp\|P02768\|ALBU | Serum albumin | 69321.6 | 30 | 1.53 |
|  | | **sum** | **212** | **100** |
| Tf@PSCOOH  **5min incubation in 55% plasma** | | | | |
| **Reference** | **Protein** | **MW [Da]** | **Peptide [Hits]** | **Norm Peak Area [%]** |
| sp\|P02787\|TRFE | Serotransferrin | 77013.67 | 106 | 59.54 |
| sp\|P04196\|HRG | Histidine-rich glycoprotein | 59540.94 | 52 | 29.61 |
| sp\|P03951\|FA11 | Coagulation factor XI | 70063.57 | 32 | 4.32 |
| sp\|P02768\|ALBU | Serum albumin | 69321.63 | 34 | 3.24 |
| sp\|P01042\|KNG1 | Kininogen-1 | 71912.14 | 11 | 1.1 |
| sp\|P00747\|PLMN | Plasminogen | 90510.23 | 17 | 1.04 |
| sp\|P01871\|IGHM | Ig mu chain C region | 49275.57 | 8 | 0.6 |
| sp\|P01024\|CO3 | Complement C3 | 187029.3 | 16 | 0.54 |
|  | | **sum** | **276** | **100** |
| **PSCOOH-Tf** | | | | |
| **Reference** | **Protein** | **MW [Da]** | **Peptide [Hits]** | **Norm Peak Area [%]** |
| sp\|P02787\|TRFE | Serotransferrin | 77013.7 | 33 | 61.19 |
| sp\|P02768\|ALBU | Serum albumin | 69321.6 | 26 | 38.81 |
|  | | **sum** | **59** | **100** |
| **PSCOOH-Tf 5min incubation in 55% plasma** | | | | |
| **Reference** | **Protein** | **MW [Da]** | **Peptide [Hits]** | **Norm Peak Area [%]** |
| sp\|P04196\|HRG | Histidine-rich glycoprotein | 59540.94 | 27 | 87 |
| sp\|P05154\|IPSP | Plasma serine protease inhibitor | 45672.76 | 2 | 3.6 |
| sp\|P02787\|TRFE | Serotransferrin | 77013.67 | 7 | 3.24 |
| sp\|P04004\|VTNC | Vitronectin | 54271.23 | 2 | 2.28 |
| sp\|P03951\|FA11 | Coagulation factor XI | 70063.57 | 8 | 1.61 |
| sp\|P02768\|ALBU | Serum albumin | 69321.63 | 6 | 1.39 |
| sp\|P01024\|CO3 | Complement C3 | 187029.3 | 3 | 0.88 |
|  | | **sum** | **55** | **100** |
| **PSCOOH 5min incubation in 55% plasma** | | | | |
| **Reference** | **Protein** | **MW [Da]** | **Peptide [Hits]** | **Norm Peak Area [%]** |
| sp\|P04196\|HRG | Histidine-rich glycoprotein | 59540.94 | 73 | 76.61 |
| sp\|P03951\|FA11 | Coagulation factor XI | 70063.57 | 29 | 8.26 |
| sp\|P01042\|KNG1 | Kininogen-1 | 71912.14 | 22 | 4.06 |
| sp\|P02768\|ALBU | Serum albumin | 69321.63 | 33 | 3.93 |
| sp\|P04004\|VTNC | Vitronectin | 54271.23 | 15 | 2.11 |
| sp\|P05154\|IPSP | Plasma serine protease inhibitor | 45672.76 | 10 | 1.53 |
| sp\|P01024\|CO3 | Complement C3 | 187029.3 | 20 | 1.07 |
| sp\|P03952\|KLKB1 | Plasma kallikrein 1 | 71322.83 | 14 | 0.92 |
| sp\|Q14624\|ITIH4 | Inter-alpha-trypsin inhibitor heavy chain H4 | 103293.2 | 13 | 0.92 |
| sp\|P00747\|PLMN | Plasminogen | 90510.23 | 13 | 0.59 |
|  | | **sum** | **252** | 100.00 |
