# Supplementary material for: Transferrin Coated Nanoparticles: Study of the Bionano Interface in Human Plasma
Source: PLoS One. 2012 Jul 19;7(7):e40685. doi: 10.1371/journal.pone.0040685 (PMC3400652; doi:10.1371/journal.pone.0040685)
Supplement: Table S2 — ProtoArray® v4.1 content and distribution by class. (DOCX) [file pone.0040685.s004.docx]

| **Class** | **No. on array** |
| --- | --- |
| Protein kinases (unique) | 268 |
| Protein kinases (including domains, splice variants, and mutants) | 776 |
| Transcription factors | 328 |
| Membrane proteins | 2,635 |
| Nuclear proteins | 2,252 |
| Signal transduction | 1,526 |
| Secreted proteins | 192 |
| Cell communication | 1,687 |
| Metabolism | 3,862 |
| Cell death | 505 |
| Protease/peptidase activity | 219 |
