# Supplementary material for: Transferrin Coated Nanoparticles: Study of the Bionano Interface in Human Plasma
Source: PLoS One. 2012 Jul 19;7(7):e40685. doi: 10.1371/journal.pone.0040685 (PMC3400652; doi:10.1371/journal.pone.0040685)
Supplement: Table S3 — The main Tf binders according to STRING algorithm. (DOCX) [file pone.0040685.s005.docx]

| **No** | **Protein** | **STRING score** |
| --- | --- | --- |
| 1 | TFRC - transferrin receptor | 0.999 |
| 2 | HFE - hemochromatosis | 0.997 |
| 3 | TFR2 - transferrin receptor 2 | 0.996 |
| 4 | HAMP - hepcidin antimicrobial peptide | 0.993 |
| 5 | CP- ceruloplasmin (ferroxidase) | 0.978 |
| 6 | ALB - albumin | 0.977 |
| 7 | MIS12 | 0.974 |
| 8 | TTR - transthyretin | 0.972 |
| 9 | PMM2 - phosphomannomutase 2 | 0.970 |
| 10 | SYNJ1 - synaptojanin 1 | 0.965 |
