# Supplementary material for: Transferrin Coated Nanoparticles: Study of the Bionano Interface in Human Plasma
Source: PLoS One. 2012 Jul 19;7(7):e40685. doi: 10.1371/journal.pone.0040685 (PMC3400652; doi:10.1371/journal.pone.0040685)
Supplement: Model S1 — Core-shell model analysis for DCS data. (DOCX) [file pone.0040685.s007.docx]

**Core-shell model analysis for DCS data.**

A simple model to analyze data for shell-coated particles was developed to get an estimation of the shell thickness. If a spherical particle, composed of an inner core of density ρ_c_, with a diameter D_c_, and a shell of density ρ_s_, and thickness D_s_, is placed in a rotating disc filled with a fluid of density ρ_f_, the particle will suffer a drag force of the form:

*Fd* =3π*D_s_*η*v* (1)

where D_s_ (D_c_+2δ) is the total diameter of the core-shell particle, η is the viscosity of the fluid and v is the settling velocity of the particle. This force will be balanced by the centrifugal force:

*Fc* =*m*ω^2^*R* (2)

where R is the distance from the particle to the axis of rotation, m is the particle mass and ω is the angular velocity of the disc (and the particle within). Considering the buoyancy and the presence of two different materials in the particle, the mass m can be written as:

*m*=$\frac{\pi}{6}$(ρ­_c_*D­_c_*^3^+ρ_s_(*D_s_-D_c_*^3^)−ρ_f_*D_s_*^3^) (3)

At equilibrium between these forces, we have:

(ρ_c_- ρ_s_)$\frac{D_{c}^{3}}{D_{s}}$+( ρ_s_- ρ_f_)D_s_^2^=$\frac{18\eta}{\omega^{2}}\frac{1}{R}\frac{dR}{dt}$ (4a)

Solving this equation for the simplest case where there is no physical shell (ρs= ρc), we obtain:

[(ρ_c_- ρ_s_)D^2^]-t=$\frac{18\eta}{\omega^{2}}$ln$\left( \frac{R_{f}}{R_{0}} \right)$ (4b)

where dR/dt is the radial velocity, t is the time elapsed while the particle moves between the initial R_0_ and the final R_f_ position and D is the measured diameter. Since all DCS measurements are calibrated for this equation in the presence of a shell one can extract a real particle diameter Ds, from the measured D using the following equation:

$\frac{(\rho_{c}- \rho_{s})}{{(\rho}_{c}- \rho_{f})}\frac{D_{c}^{3}}{D_{s}}+\frac{(\rho_{s}-\rho_{f})}{(\rho_{c}-\rho_{f})}D_{s}^{2}=D^{2}$ (5)

Generally, our DCS results for NP-protein complexes showed a particle size distribution with a main peak (100%) related to the monomeric particle population. From the apparent measured diameter of these peaks we extracted the shell thickness, δ, by knowing ρ_c_, ρ_f_, ρ_s_ and D_c_. In particular, D_c_ is set to the value obtained for the bare NPs in buffer and ρ_c_ is the density of the material. Actually, ρ_f_ should be considered as a function of the radius R but it is substituted with an effective quantity, which is its mean value between R_0_ and R_f_. In our case, a source of uncertainty for the quantitative determination of the shell thickness is the choice for the shell density since we do not have experimental values for the hydration degree and the actual conformation of the adsorbed proteins. Moreover, we have a mixture of proteins (see gel in Figure S4c) with a wide range of Mws S6whose density values can differ significantly. The established mean density value for hydrated protein crystals is 1.23 g/cm^3^, while for blood plasma it is 1.025g/ml, thus we considered that the density of the protein shell could be an intermediate value between these and took their mean, which is 1.125g/ml.
